# Evidence on the Bioaccessibility of Glucosinolates and Breakdown Products of Cruciferous Sprouts by Simulated In Vitro Gastrointestinal Digestion

**DOI:** 10.3390/ijms222011046

**Published:** 2021-10-13

**Authors:** Ángel Abellán, Raúl Domínguez-Perles, Cristina García-Viguera, Diego A. Moreno

**Affiliations:** Laboratory of Phytochemicals and Healthy Foods (LabFAS), Group on Quality, Safety, and Bioactivity of Plant Foods (CEBAS-CSIC), Campus Universitario de Espinardo, 25, 30100 Murcia, Spain; avictorio@cebas.csic.es (Á.A.); cgviguera@cebas.csic.es (C.G.-V.); dmoreno@cebas.csic.es (D.A.M.)

**Keywords:** brassica, sprouts, sulfur-based compounds, isothiocyanates, indoles, bioaccessibility

## Abstract

Cruciferous vegetables are gaining importance as nutritious and sustainable foods, rich in phytochemical compounds such as glucosinolates (GSLs). However, the breakdown products of these sulfur-based compounds, mainly represented by isothiocyanates (ITC) and indoles, can contribute to human health. In the human digestive system, the formation of these compounds continues to varying extents in the different stages of digestion, due to the contact of GSLs with different gastric fluids and enzymes under the physicochemical conditions of the gastrointestinal tract. Therefore, the aim of the present work was to uncover the effect of gastrointestinal digestion on the release of glucosinolates and their transformation into their bioactive counterparts by applying a simulated in vitro static model on a range of brassica (red radish, red cabbage, broccoli, and mustard) sprouts. In this sense, significantly higher bioaccessibility of ITC and indoles from GSLs of red cabbage sprouts was observed in comparison with broccoli, red radish, and mustard sprouts, due to the aliphatic GSLs proportion present in the different sprouts. This indicates that the bioaccessibility of GSLs from *Brasicaceae* sprouts is not exclusively associated with the initial content of these compounds in the plant material (almost negligible), but also with the release of GSLs and the ongoing breakdown reactions during the gastric and intestinal phases of digestion, respectively. Additionally, aliphatic GSLs provided higher bioaccessibility of their corresponding ITC in comparison to indolic and aromatic GSLs.

## 1. Introduction

In recent decades, dietary patterns have been recognized as a key factor contributing to human health, with the proportion of plant-based foods to achieve healthy diets being noticed as especially important. As a result, several studies have focused on the bioactive constituents (nutrients and non-nutrients) present in foods, with special attention being devoted to fruits and vegetables that have been associated with such beneficial effects on health. Indeed, among the diverse range of foods integrated into regular diets, plant-based foods have been suggested as sources of the bioactive nutrients and phytochemical compounds responsible for effects on health [1]. With respect to plant-based foods, the inclusion of cruciferous varieties in dietary recommendations has intensified, as they provide particularly health-promoting nutrients and compounds, mainly represented by micronutrients (vitamins and minerals) and bioactive phytochemicals (e.g., glucosinolates (GSLs)). This composition has been associated with the prevention of several non-communicable diseases, such as inflammatory and tumoral processes, as well as radical scavenging activities and the prevention of diverse pathological metabolic processes [2].

In this context, cruciferous sprouts, namely broccoli (*Brassica oleracea* var. *italica* L. cv. ‘Calabrese’), red cabbage (*Brassica oleracea* L. var. *capitata* f. rubra), red radish (*Raphanus sativus* var. *sativus* L. cv. ‘Rambo’), and white mustard (*Sinapis alba* L.), have been reported to be valuable dietary sources of GSLs, the characteristic bioactive phytochemicals present in *Brassicaceae* foods [3,4], mainly represented by aplihatic glucoraphanin (in broccoli and cabbage) and glucoraphenin (in radish), aromatic glucosinalbin (mustard), and the characteristic indolic GSLs, which includes glucobrassicin and its hydroxy- and methoxy-derivatives, present in all ed varieties, each of them featured by specific biological traits [5,6].

The biological activity of GSLs relies on their hydrolysis catalyzed by myrosinase. This is an esterase present within vacuoles in vegetable cells that is released due to mechanical damage or other biotic stress events (e.g., chewing, hydric deficit, and/or parasite attack) [7]. The hydrolysis of GSLs by myrosinase generates bioactive molecules, such as isothiocyanates (ITC) and indoles [8]. The biological benefits described so far regarding both ITC and indoles are due to their ability to react with amino groups, sulfhydryl (-SH) radicals, and disulphide bonds present in proteins and amino acids, as well as with the phenolic group of tyrosine residues of proteins [9,10].

**Figure 1:**
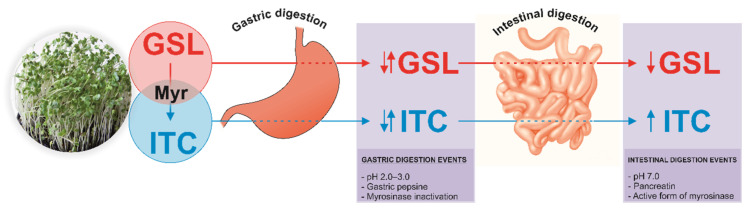
Schematic representation of the factors involved in the stability of glucosinolates (GSLs) and isothiocyanates (ITCs) during gastrointestinal digestion. Myr, myrosinase.

The bioactivity exerted by ITCs and indoles is dependent on their bioavailability rate, which in turn is conditioned by the bioaccessibility of GSLs and their transformation products into the intestinal tract. The so-called bioaccessibility represents the release of a given compound from the food matrix during gastrointestinal digestion and its stability under the physicochemical conditions of the gastrointestinal tract. The efficiency of this process depends on both the physical features of the food matrix and the chemical characteristics of the bioactive compounds of interest [11]. Different aspects, such as pH dynamics, food matrix, and enzymatic activity, can modify the content of bioactive compounds present in the food matrix [12], requiring in-depth characterizations of the different matrices and analytes (Figure 1).

Nonetheless, at the present time, there remains a gap in knowledge regarding the impact of gastrointestinal digestion on the bioactive compounds of edible sprouts, the presence of which in diets is rising in parallel with their recognition as healthy foods.

On the basis of these antecedents, the present work is aimed at uncovering the bioaccessibility of GSLs and ITCs present in different cruciferous sprouts (broccoli, red radish, mustard, and red cabbage). In addition, we aim to shed light on the relative contribution of the separate stages of the digestive process to the final concentration of GSLs and ITCs in the digestates, considering the release of GSLs and/or their transformation into their bioactive derivatives during the gastric and intestinal phases of gastrointestinal digestion individually.

## 2. Results and Discussion

### 2.1. Intact Dlucosinolates and Isothiocyanates in Cruciferous Sprouts

**Figure 2:**
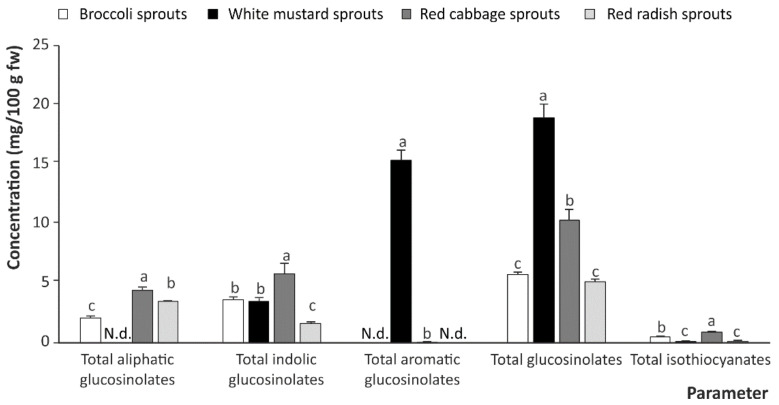
Total content of glucosinolates in undigested cruciferous sprouts. Mean values ± SD (*n* = 3) for each family of glucosinolates with different lowercase letters are significantly different at *p* < 0.01 according to the analysis of variance (ANOVA) and Tukey’s multiple range test. N.d., not detected.

The total contents of intact GSLs and ITCs in the studied cruciferous sprouts, before in vitro digestion, evidenced the predominance of GSLs—total concentration of which decreased as follows: white mustard (18.99 mg/100 g of fresh weight (fw)) > red cabbage (10.39 mg/100 g fw) > broccoli and red radish (5.50 mg/100 g fw, on average) (Figure 2)—relative to the ITC content, which was negligible in the intact plant material (0.46 mg/g fw, on average, in all sprouts) (Figure 2).

When analyzing the contribution of the different types of GSL (aliphatic, indolic, and aromatic GSLs), the profiles were found to be very closely dependent on the sprout species considered. Therefore, regarding total aliphatic GSLs, the red cabbage displayed the highest concentration (4.49 mg/100 g fw), followed by red radish (3.54 mg/100 g fw), and broccoli (2.16 mg/100 g fw). Aliphatic GSLs were almost absent in white mustard sprouts (Figure 2). These results are in good agreement with previous works assessing the GSL contents of sprouts of the Brassicaceae family describing a similar profile [13,14]. Concerning indolic GSLs, in general, this group exhibited concentrations ~30% higher than aliphatic GSLs, on average, with the exception of red radish sprouts, in which the concentration of indolic GSLs was lower than the level found for aliphatic GSLs. Additionally, significant differences were recorded between the cruciferous species analyzed. The highest concentration was found in red cabbage sprouts (5.85 mg/100 g fw) followed by broccoli and white mustard sprouts (3.61 mg/100 g fw, on average). The lowest concentration of indole GSLs corresponded to red radish sprouts (1.65 mg/100 g fw) (Figure 2), again showing results that were in agreement with previous reports in the literature [13,14]. Finally, aromatic GSLs were only found in white mustard sprouts in quantifiable amounts (15.43 mg/100 g fw), followed by traces in red radish sprouts (Figure 2), which is in agreement with the information described in recent years [15].

The individual compounds belonging to each class of GSLs were assessed. The different compounds were identified with respect to their retention time, parent mass, and specific fragmentation pattern in comparison with authentic standards and information available in the literature (Table 1) [16,17].

When profiling aliphatic GSLs, the individual compounds identified were found to be closely dependent on the variety considered. Therefore, this GSL class was mainly represented by glucoraphanin and glucoerucin (GR and GE, respectively) in red cabbage sprouts; glucoerucin, glucoraphanin, and glucoraphenin (GRE) in red radish sprouts; and glucoraphanin in broccoli sprouts. Aliphatic GSLs in mustard sprouts were almost completely absent or, present at concentrations lower than the limit of detection of the analytical technique (Table 2).

Again, the results retrieved on the GSL profiles of red cabbage, red radish, broccoli, and mustard sprouts are in good agreement with previous characterizations [13,14], associating them with biological advantages when incorporated into the regular diet, thus demonstrating the healthy composition of the brassica sprouts characterized in the present work.

Additionally, glucobrassicin and its related forms methoxy-glucobrassicin, hydroxy-glucobrassicin, and neo-glucobrassicin (MeGB, OHGB, and NeoGB, respectively) were identified as representative compounds of indolic GSLs. When profiling this family of GSLs in the different sprouts considered, significant differences between them were observed. Thus, MeGB was the predominant indolic GSL in all sprouts, followed by NeoGB in mustard and broccoli sprouts, and OHGB in red cabbage sprouts, which is in accordance with previous results reported in other works [18,19]. Finally, aromatic GSLs were exclusively represented by glucosinalbin, which was the predominant GSL in white mustard sprouts, followed by radish sprouts, which also exhibited the presence of these GSLs, although at a significantly lower concentration, which is in good agreement with currently available descriptions in the literature [15].

### 2.2. Breakdown Products from Sulfur-Based Glucosinolates after In Vitro Digestion of Cruciferous Sprouts

Taking into account the content of intact GSLs in fresh, undigested plant material, the hydrolysis of these compounds towards the generation of their bioactive counterpart products (ITC and indoles) during the different stages of gastrointestinal digestion (in vitro digestion) was analyzed. As expected, the intact GSLs were not detected in any of the digestates obtained following gastric, intestinal, and gastrointestinal digestion. This result is in contrast with the descriptions by Fernández-León et al. (2016), who found amounts of GSLs in quantifiable concentrations in gastrointestinal digestates of broccoli florets (although, in agreement with the results found in the present work, these concentrations were lower than the concentration of these compounds in undigested plant material) [20]. The total absence of GSLs in the gastrointestinal digestates could be explained by the specific physical properties of sprouts relative to adult plant material, which confers a differential capacity to resist the gastric conditions (low pH, pepsin activity, and the presence of epithiospecifier proteins, among others) [20,21]. Despite this, an augmentation of ITCs was observed during the digestion stages in comparison with fresh, undigested, plant material (red cabbage, mustard, broccoli, and red radish sprouts.

#### Influence of Gastric, Intestinal, and Gastrointestinal Digestion

**Figure 3:**
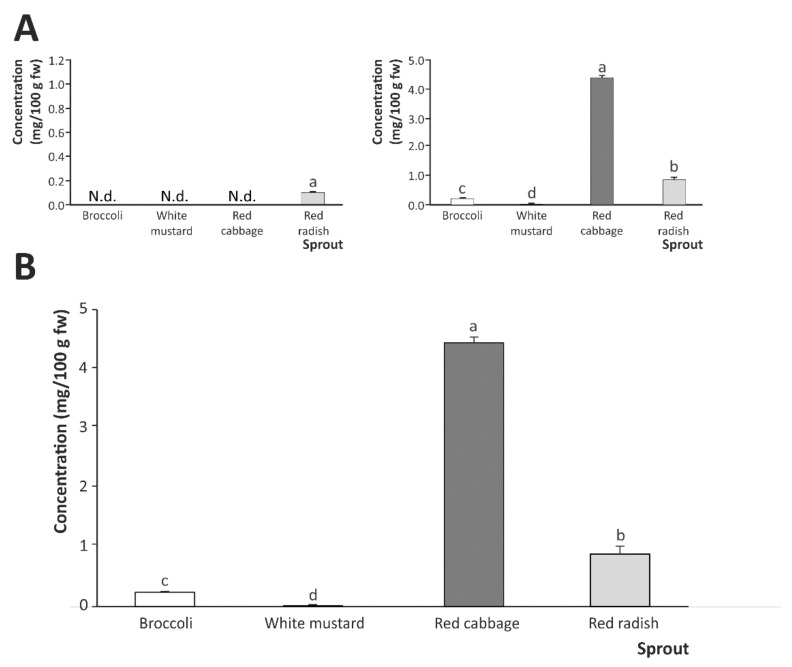
Total content of ITCs and indoles in the digestates after gastric phase (**A**, left bar-plot), intestinal phase (**A**, right bar-plot), and gastrointestinal phase (**B**). Results are expressed as mean ± SD (*n* = 3). Within each plot, bars with different lowercase letters are significantly different at *p* < 0.01 according to the analysis of variance (ANOVA) and Tukey’s multiple range test. N.d., not detected.

The capacity of the simulated gastric digestion to generate hydrolysis products from GSLs was analyzed, both when considering gastric digestion separately and in sequential combination with intestinal in vitro digestion (gastrointestinal phase). The results obtained showed a limited extraction of GSLs, ITCs, and indoles from the plant material by the gastric digestion, producing ITCs, as can be seen in Figure 3A (left bar-plot). In this regard, only digestates from red radish sprouts exhibited concentrations higher than the LOQ (0.10 mg/100 g fw). This result is in good agreement with previous descriptions in the literature regarding the capacity of gastric digestion to extract ITCs and indoles from brassicas after dietary intake [22,23]. In addition, the low pH featured in the simulated gastric fluid (SGF) might promote the formation of nitriles and epionitriles instead of ITCs [24,25]. However, when the digestates from the gastric phase (chymo) were exposed to intestinal physico-chemical conditions and the enzymatic activity characterizing this stage, a significant increase of bioaccessible ITCs was observed (Figure 3A, right bar-plot) relative to the results retrieved following gastric digestion. Thus, the highest concentration of ITCs corresponded to red cabbage (4.41 mg/100 g fw), followed by red radish (0.86 mg/100 g fw) and broccoli (0.20 mg/100 g fw), while the concentration of ITCs in the intestinal digestates obtained from mustard sprouts was almost negligible. The specific extraction of ITCs from brassica foods during intestinal digestion fits well with previous studies [26,27].

These results make it possible to understand the actual contribution of each digestion stage (gastric and intestinal) to the intestinal concentration of ITCs during the whole gastrointestinal digestion process (Figure 3B). Indeed, the amounts of total ITCs reached following gastrointestinal digestion were very similar to those obtained when considering intestinal digestion separately, with the exception of red radish. Therefore, the highest values corresponded to red cabbage sprouts, which exhibited the highest content of hydrolysis products (ITCs) following gastrointestinal digestion (4.41 mg/100 g fw), followed by red radish sprouts (0.88 mg/100 g fw), broccoli sprouts (0.24 mg/100 g fw), and, finally, mustard sprouts (0.01 mg/100 g fw) (Figure 3B). These results highlight the disproportionality between the concentration of GSLs in the plant material and the generation of ITCs, which can be explained by the different chemical structures associated with the diverse groups of GSLs (aliphatic, indole, and aromatic), and depending on the amino acid group linked to the main structure [28].

When assessing digestates with respect to their ITC and indole profiles in order to shed light on the specific bioaccessibility traits associated with aliphatic, indolic, and aromatic GSLs, it was found that sulforaphane (SFN) and iberin (IB), hydrolysis products from GR and GI, respectively, were present in the gastrointestinal digestates of all sprout varieties characterized in the present work. The highest concentration of SFN corresponded to the digestates of red cabbage sprouts (0.204 mg/100 g fw), surpassing the level of the digestates of broccoli by 36.8% and those of red radish and mustard sprouts by 93.1%, on average. For IB, again, the digestates of red cabbage sprouts exhibited the highest concentration (4.190 mg/100 g fw), followed by digestates of red radish (an 80.0% lower) and broccoli (a 98.4% lower) sprouts, while this ITC was almost absent in the digestates of white mustard sprouts (Table 2).

Similarly, indole-3-carbinol (I3C), an indole formed by the hydrolysis of GB catalyzed by esterases, was present in red radish and broccoli sprouts’ digestates in similar concentrations (0.043 mg/100 g fw, on average), followed by digestates of red cabbage sprouts (32.6% lower). This compound was not detected in the digestates of white mustard’s sprouts despite the GB content of this variety (Table 2). These data suggest the influence of the amino acid precursor and chemical properties of GSLs on the bioaccessibility of the own GSLs and hydrolysis products, as well as the relevance of additional features of the food matrix, which could strongly condition the effect of gastrointestinal digestion on the release and stability/hydrolysis of the compounds of interest [29].

The analysis of the relative contribution of the separate phases of gastrointestinal digestion indicated the limited capacity of gastric digestion to extract and hydrolyze glucosinolates, further confirming the results obtained regarding total GSLs, ITC, and indoles (Figure 2 and Table 3). Thus, the potential of gastric digestion to extract and hydrolyze GSLs was very limited, appearing restricted to sulforaphene and indole-3-carbinol (SFE and I3C, respectively). Moreover, the specific physicochemical properties of the food matrix seemed to also be relevant to the efficiency of this stage in extracting compounds, as this was restricted to broccoli and red radish sprouts (Table 3). On the other hand, and also confirming results mentioned previously regarding total compounds, the intestinal phase was the most relevant for the bioaccessibility of the breakdown products of GSLs, especially with respect to SFN and IB.

**Figure 4:**
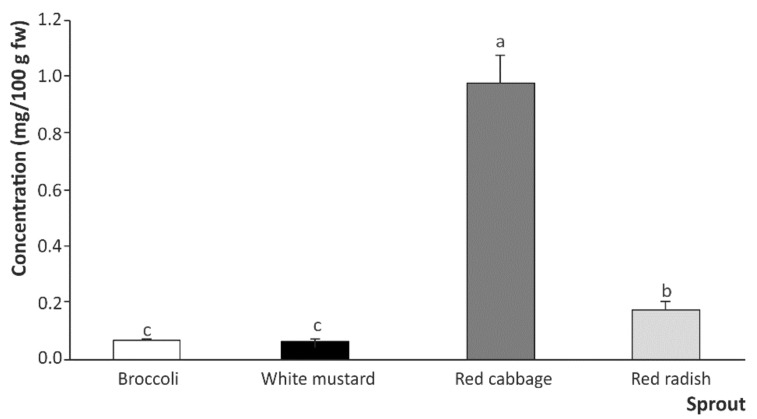
Total content of ITCs and indoles in the digestates after direct intestinal digestion on plant material. Results are expressed as mean ± SD (*n* = 3). Bars with different lowercase letters are significantly different at *p* < 0.01 according to the analysis of variance (ANOVA) and Tukey’s multiple range test.

The results obtained regarding the predominant relevance of intestinal digestion to the formation of ITC and indoles from the GSLs present in brassica sprouts as a result of the dietary intake prompted us to evaluate the intestinal digestion of fresh material, without the interference of gastric digestion conditions. This approach allowed us to describe the predominant effect of the intestinal digestion phase with respect to the extraction of GSLs and their hydrolysis towards bioactive ITCs and indoles. Nonetheless, despite the trend regarding the features of different sprout types when evaluated as sources of bioaccessible ITCs and indoles (red cabbage >> red radish > broccoli > white mustard), when applying the physicochemical and enzymatic conditions featured in intestinal digestion directly to the raw plant, the concentrations of total ITCs and indoles obtained (0.07, 0.07, 0.98, and 0.18 mg/g fw, respectively, Figure 4) were much lower compared to those obtained from the gastric digestates (0.20, <LOQ, 4.41, and 0.86 mg/g fw, respectively, Figure 2).

In the same way, this result was confirmed when profiling the individual compounds present in the digestates obtained as a result of the direct intestinal digestion of raw brassica sprouts, with the exception of SFE, which was degraded during the intestinal phase in the context of sequential gastric and intestinal digestion (Table 3 and Table 4). In this regard, the highest concentration of bioaccessible SFN, IB, and I3C upon direct intestinal digestion of the plant material corresponded to red cabbage sprouts (0.064, 0.840, and 0.076 mg/100 g fw, respectively), which were in any case, and for all sprouts, lower than the values obtained when intestinal digestion was developed on the basis of the results of the gastric stage.

This provided information on the essential preparatory role of gastric digestion in achieving the concentration described in the gastrointestinal digestates (tentatively attributed to the effect of the acid conditions of SGF and gastric pepsine on the food matrix, improving the efficiency of intestinal digestion). Indeed, the sequential gastrointestinal workflow during digestion allows the liberation of GSLs from the food matrix and their transformation into breakdown products during intestinal digestion in a more efficient way, thereby taking advantage of the bioactive compounds obtained by the ingestion of brassica sprouts and their benefits for health with respect to diverse pathophysiological processes [30].

## 3. Materials and Methods

### 3.1. Chemical and Reagents

The standards of GR, GRE, GI, GB, OHGB, MeGB, NeoGB, and GSB were purchased from Phytoplan GmbH (Heidelberg, Germany). The standards of SFN and SFE were obtained from Santa Cruz Biotechnology (Dallas, TX, USA) and the standards of iberin and I3C were purchased from Biorbyt LTD (Cambridge, UK) and LKT Laboratories (St. Paul, MN, USA), respectively. Acetic acid, hydrochloric acid, and ammonium acetate were purchased from Panreac (Barcelona, Spain). Methanol for hydromethanolic extractions and all LC-MS grade solvents (acetonitrile and acetic acid) was provided by J.T. Baker (Philipsburg, NJ, USA). All water employed in the extraction and the chromatographic analysis was treated with a Milli-Q water purification system (Millipore, Bedford, MA, USA).

### 3.2. Plant Material

All brassica seeds corresponding to broccoli (*Brassica olreacea* var. *italica* L. cv. Calabrese), red radish (*Raphanus sativus* var. *sativus* L. cv. Rambo), white mustard (*Sinapis alba* L.), and red cabbage (*Brassica oleracea*, var. *capitata* f. *rubra*) were provided by Intersemillas S.A. (Loriguilla, Valencia, Spain). For germination purposes, seeds were decontaminated with 1% bleach in distilled water and aerated for 24 h. Afterward, seeds were transferred to trays with cellulose and kept in darkness for 48 h to stimulate stem elongation. Then, sprouts were placed in a growth chamber under controlled conditions (light/darkness photoperiod 18/6 h, temperature 24/18 °C, and relative humidity 60/80%) for 6 days. At the end of the growing period, the sprouts were collected, frozen at −80 °C for 24 h, freeze-dried, and ground to a fine powder. Preprocessed samples were stored at room temperature, protected from light and humidity, until analysis.

### 3.3. Processing Cruciferous Sprouts by a Simulated In Vitro Static Digestion Method

Gastric, intestinal, and gastrointestinal digestion were performed on the brassica sprout powder (500 mg) following a previously described methodology [31,32]. Briefly, for the development of the gastric digestion, the samples (500 mg) were mixed with 15 mL of simulated gastric fluid (SGF) stock electrolyte solution prepared according to the information referred to in Table 3. In brief, pepsin enzyme (EC 3.4.23.1) was prepared by dissolving in SGF until a final concentration of 2000 U/mL, and stirred continuously at 52 oscillations per min, for 2 h, at 37 °C in a thermal water bath (Unitronic™ Vaiven, J.P. SELECTA, Barcelona, Spain). The final pH was adjusted by adding 1 M HCl to obtain a pH 3.0 buffer. Triplicate samples were assayed, and the reaction was ended by adding sodium hydroxide solution (0.2 M).

To simulate the intestinal and gastrointestinal digestion, a simulated intestinal fluid (SIF) was prepared by mixing enzyme solution (pancreatin (EC 232-468-9) and pancreatic lipase (EC 3.1.1.3)) according to the information provided in Table 5. The enzyme concentrations were 100 U/mL (trypsin activity) and 64 U/mL (lipase activity) for pancreatin and 2000 U/mL for pancreatic lipase. Frozen porcine bile salts were added to achieve the final concentration of 10 mM. The pH of the SIF was adjusted at 8.0 by adding the required volume of 1 M NaOH. Again, the intestinal digestion of both the sprout powder and the gastric chime were performed for 2 h at 37 °C in a thermal bath under continuous stirring.

After gastric, intestinal, and gastrointestinal digestion, the samples were centrifuged at 2000 rpm, for 5 min, at 4 °C to separate the soluble or bioaccessible fraction and the residual fraction. The bioaccessible fraction was saved and frozen at −80 °C and lyophilized. For the extraction of GSLs, ITC, and indoles, the lyophilized samples were then dissolved in 1 mL of MeOH/deionized water (70:30, *v*/*v*), sonicated 30 min, centrifuged at 2000 rpm, for 5 min, and filtered through a 0.45 μm filter (Millipore, MA, USA)

### 3.4. UHPLC-ESI-QqQ-MS/MS Analysis of Analytical Extracts and Digestates of Brassica Sprouts

The chromatographic separation of GSLs and isothiocyanates present in the analytical extracts and digestates was performed using a UHPLC coupled with a 6460 triple quadropole-MS/MS (Agilent Technologies, Waldbronn, Germany) and a Zorbax Eclipse Plus C18 column (2.1 × 50 mm, 1.7 µm). Chromatographic conditions were set according to Domínguez-Perles et al. (2014) and Baenas et al. (2017) [16,17], with minor modifications. Briefly, the mobile phases employed were deionized water/ammonium acetate 13 mM (pH 4) (99.99:0.01, *v*/*v*) (solvent A) and acetonitrile/acetic acid (99.99:0.1, *v*/*v*) (solvent B). The chromatographic separation of the target compounds was performed according to the following gradient (time (minutes), %B): (0, 12%); (0.21, 30%); (1.00, 30%); (1.01, 52%); (4.00, 100%); (7.50, 100%); (7.51, 12%). The injection volume and flow rate were 10 µL and 0.3 mL/min, respectively. Data were acquired employing MassHunter software version B.08.00 (Agilent). The identification and quantification of the separate glucosinolates and isothiocyanates were performed resorting to the qualitative and quantitative transitions, and calibration curves of authentic standards freshly prepared each day of analysis (Table 1).

### 3.5. Statistical Analysis

Results are presented as means ± SD (*n* = 3). A paired t-test was developed to compare two measurements at different time-points, and analysis of variance (ANOVA) and Tukey’s multiple range tests were carried out to compare three or more conditions, both in accordance with the normal distribution of the parameters determined. All statistical analyses were performed using SPSS 25.0 software (LEAD Technologies, Inc., Chicago, IL, USA). The level of statistical significance was set at *p* < 0.01.

## 4. Conclusions

The major results obtained in the present work demonstrate that there is no direct relationship between intact GSLs content and the final concentration of ITCs achieved in the intestinal tract due to the hydrolysis reactions developed as a result of gastrointestinal digestion. Moreover, the data showed a clear influence of the amino acid group linked to the GSLs on bioaccessibility. Thus, the breakdown products of aliphatic GSLs were the group that exhibited the highest bioaccessibility, followed by those from indolic GSLs. In addition to the chemical properties of the separate GSLs and their breakdown products, the final concentration appeared to additionally be closely related to the composition and physicochemical properties of the specific dietary source under consideration. In this sense, red cabbage sprouts exhibited the greatest quantity of ITC, when compared with red radish, broccoli or mustard sprouts. This result further encourages the investigation of the bioaccessibility and bioavailability of the GSLs, ITC, and indoles from rich dietary sources such as the sprouts of cruciferous varieties to support their value as dietary sources of these interesting biologically active compounds.

## Figures and Tables

**Table 1:** UHPLC-ESI-QqQ-MS/MS parameters for the identification and quantification of glucosinolates and isothiocyanates present in broccoli, red radish, white mustard, and red cabbage extracts.

Compound	MRM Quantitative Transition	MRM Qualitative Transition	Fragmentation (V)	Collision Energy (eV)	ESI Mode
Aliphatic glucosinolates					
GR	436.0 > 97.0	436.0 > 372.0	90	25	Negative
GRE	434.0 > 97.0	434.0 > 259.0	90	25	Negative
GE	420.0 > 97.1	420.0 > 259.0	60	20	Negative
GI	422.0 > 357.7	422.0 > 259.0	100	26	Negative
GN	372.0 > 97.0	372.0 > 259.0	90	25	Negative
Indolic glucosinolates					
GB	447.2 > 97.0	447.2 > 259.0	80	20	Negative
MeGB ^Y^	477.0 > 97.0	477.0 > 259.0	90	25	Negative
OHGB ^Y^					Negative
NeoGB	463.0 > 97.0	463.0 > 259.0	90	25	Negative
Aromatic glucosinolates					
GNS	422.0 > 97.0	422.0 > 259.0	90	25	Negative
GT	408.0 > 97.0	N.d.	90	25	Negative
GSB	424.4 > 97.0	424.10 > 259.0	90	25	Negative
Isothiocyanates					
SFN	178.0 > 114.0	178.0 > 95.0	74	4	Positive
SFE	176.0 > 114.0	N.d.	75	20	Positive
E	141.0 > 59.0	161.0 > 70.0	70	6	Negative
IB	164.0 > 105.0	N.d.	90	6	Positive
Indoles					
I3C	130.0 > 77.0	247.0 > 130.0	70	25	Positive

GR, glucoraphanin; GRE, glucoraphenin; GE, glucoerucin; GI, glucoiberin; GN, gluconapin; GB, glucobrassicin; MeGB, Methoxy-glucobrassicin; OHGB, hydroxy-glucobrassicin; NeoGB, Neo-glucobrassicin; GNS, gluconasturtin; GT, glucotrapeolin; GSB, glucosinalbin; SFN, sulforaphane; SFE, sulforaphane; E, erucin; IB, iberin; I3C, Indole-3-carbinol; MRM, multiple reaction monitoring; ESI, electrospray ionization; N.d., not determined. ^Y^ coeluting compounds.

**Table 2:** Content (mg/100 g fw) of individual glucosinolates and glucosinolate breakdown products (isothiocyanates and indoles) in the studied sprouts. (control).

Sprouts	Glucosinolates								Isothiocyanates			Indoles
	Aliphatic			Indolic				Aromatic				
	GR	GRE	GE	GB	MeBG	OHGB	NeoGB	GSB	SFN	SFE	IB	I3C
Broccoli	2.16 ± 0.13 a	N.d.	N.d.	0.78 ± 0.39 c	1.61 ± 0.02 b	0.46 ± 0.08 b	0.80 ± 0.15 c	N.d.	0.235 ± 0.001 a	N.d.	0.125 ± 0.002 a	0.196 ± 0.003 b
White mustard	N.d.	N.d.	N.d.	1.08 ± 0.11 bc	1.30 ± 0.19 c	N.d.	1.18 ± 0.05 a	15.43 ± 0.81 a	0.0020 ± 0.001 b	N.d.	N.d.	0.150 ± 0.005 c
Red cabbage	2.45 ± 0.14 a	N.d.	2.04 ± 0.22 a	1.13 ± 0.32 a	2.29 ± 0.42 a	1.40 ± 0.09 a	1.02 ± 0.02 b	0.05 ± 0.01 b	0.231 ± 0.004 a	N.d.	0.19 ± 0.01 b	0.534 ± 0.012 a
Red radish	0.30 ± 0.04 b	0.97 ± 0.02 a	2.26 ± 0.06 a	0.16 ± 0.01 d	1.48 ± 0.18 bc	N.d.	N.d.	N.d.	0.025 ± 0.002 b	0.077 ± 0.002 a	N.d.	0.045 ± 0.002 d

GR, glucoraphanin; GRE, glucoraphenin; GE, glucoerucin; GI, glucoiberin; GN, gluconapin; GB, glucobrassicin; MeGB, Methoxy-glucobrassicin; OHGB, hydroxy-glucobrassicin; NeoGB, Neo-glucobrassicin; GNS, gluconasturtin; GT, glucotrapeolin; GSB, glucosinalbin; SFN, sulforaphane; SFE, sulforaphane; E, erucin; IB, iberin; I3C, Indole-3-carbinol. Mean SD (*n* = 3) followed by different lowercase letters are significantly different at *p* < 0.01 according to the analysis of variance (ANOVA) and Tukey’s multiple range test. N.d., not detected.

**Table 3:** Content of bioaccessible individual isothiocyanates and indoles (mg/100 g fw) extracted from different plant materials due to gastric, intestinal, and gastrointestinal digestion.

Sprout	Glucosinolate Breakdown Products			
	SFN	SFE	IB	I3C
Gastrointestinal digestion				
Broccoli	0.129 ± 0.015 b	N.d.	0.070 ± 0.001 c	0.040 ± 0.004 a
White mustard	0.013 ± 0.002 c	N.d.	N.d.	N.d.
Red cabbage	0.204 ± 0.004 a	N.d.	4.190 ± 0.080 a	0.046 ± 0.004 a
Red radish	0.014 ± 0.001 c	N.d.	0.840 ± 0.100 b	0.029 ± 0.001 b
Gastric digestion				
Broccoli	N.d.	N.d.	N.d.	0.048 ± 0.001 b
White mustard	N.d.	N.d.	N.d.	N.d.
Red cabbage	N.d.	N.d.	N.d.	N.d.
Red radish	N.d.	0.039 ± 0.001 a	N.d.	0.063 ± 0.006 a
Intestinal (theoretical) digestion				
Broccoli	0.129 ± 0.015 b	N.d.	0.070 ± 0.001 c	N.d.
White mustard	0.013 ± 0.002 c	N.d.	N.d.	N.d.
Red cabbage	0.204 ± 0.012 a	N.d.	4.190 ± 0.080 a	0.046 ± 0.004 a
Red radish	0.014 ± 0.001 c	N.d.	0.890 ± 0.050 b	N.d.

SFN, sulforaphane; SFE, sulforaphene; IB iberin; I3C, indole-3-carbinol. Mean SD (*n* = 3) followed by different lowercase letters are significantly different at *p* < 0.01 according to the analysis of variance (ANOVA) and Tukey’s multiple range test. N.d., not detected.

**Table 4:** Content of bioaccessible individual isothiocyanates and indoles (mg/100 g fw) extracted from the different sprouts as a result of the intestinal (theoretical) digestion developed directly from the raw plant material (broccoli, white mustard, red cabbage, and red radish sprouts).

Sprouts	Glucosinolate Breakdown Products			
	SFN	SFE	IB	I3C
Broccoli	0.026 ± 0.001 b	N.d.	0.007 ± 0.001 c	0.035 ± 0.001 c
White mustard	0.006 ± 0.001 c	N.d.	N.d.	0.061 ± 0.001 b
Red cabbage	0.064 ± 0.001 a	N.d.	0.840 ± 0.092 a	0.076 ± 0.004 a
Red radish	0.006 ± 0.001 c	0.055 ± 0.014 a	0.116 ± 0.002 b	0.063 ± 0.006 b

SFN, sulforaphane; SFE, sulforaphene; IB iberin; I3C, indole-3-carbinol. Mean SD (*n* = 3) followed by different lowercase letters indicate significant difference at *p* < 0.01 according to the analysis of variance (ANOVA) and Tukey’s multiple range test. N.d., not detected.

**Table 5:** Composition of simulated gastric and intestinal fluids (SGF and SIF, respectively).

Consituent	Concentration of SGF, PH 3 (mmol L^−1^)	Concentration of SIF, PH 7 (mmol L^−1^)
Potassium chloride (KCl)	6.90	6.80
Potassium dihydrogenphosphate (KH_2_PO_4_)	0.90	0.80
Sodium hydrogen carbonate (NaHCO_3_)	25.00	85.00
Sodium chloride (NaCl)	47.20	38.40
Magnesium chloride (MgCl_2_)	0.10	0.33
Ammonium carbonate ((NH_4_)CO_3_)	0.50	
